# Adversarial Robustness Enhancement for Deep Learning-Based Soft Sensors: An Adversarial Training Strategy Using Historical Gradients and Domain Adaptation

**DOI:** 10.3390/s24123909

**Published:** 2024-06-17

**Authors:** Runyuan Guo, Qingyuan Chen, Han Liu, Wenqing Wang

**Affiliations:** School of Automation and Information Engineering, Xi’an University of Technology, Xi’an 710048, China; guorunyuan@xaut.edu.cn (R.G.); 2220320106@stu.xaut.edu.cn (Q.C.); wangwenqing@xaut.edu.cn (W.W.)

**Keywords:** adversarial attack and defense, deep learning, soft sensors

## Abstract

Despite their high prediction accuracy, deep learning-based soft sensor (DLSS) models face challenges related to adversarial robustness against malicious adversarial attacks, which hinder their widespread deployment and safe application. Although adversarial training is the primary method for enhancing adversarial robustness, existing adversarial-training-based defense methods often struggle with accurately estimating transfer gradients and avoiding adversarial robust overfitting. To address these issues, we propose a novel adversarial training approach, namely domain-adaptive adversarial training (DAAT). DAAT comprises two stages: historical gradient-based adversarial attack (HGAA) and domain-adaptive training. In the first stage, HGAA incorporates historical gradient information into the iterative process of generating adversarial samples. It considers gradient similarity between iterative steps to stabilize the updating direction, resulting in improved transfer gradient estimation and stronger adversarial samples. In the second stage, a soft sensor domain-adaptive training model is developed to learn common features from adversarial and original samples through domain-adaptive training, thereby avoiding excessive leaning toward either side and enhancing the adversarial robustness of DLSS without robust overfitting. To demonstrate the effectiveness of DAAT, a DLSS model for crystal quality variables in silicon single-crystal growth manufacturing processes is used as a case study. Through DAAT, the DLSS achieves a balance between defense against adversarial samples and prediction accuracy on normal samples to some extent, offering an effective approach for enhancing the adversarial robustness of DLSS.

## 1. Introduction

Complex industrial manufacturing processes face significant challenges in sensing key primary variables in complicated environments, which are usually measured by corresponding hardware sensors [1]. These environments are characterized by high temperatures, corrosion, and strong magnetic fields, among other challenging conditions, thereby making it difficult for hardware sensors to operate stably over extended periods of time [2,3]. Therefore, soft-sensor methods have been extensively studied and deployed in recent years. Soft-sensor methods establish models by utilizing mathematical mapping relationships between easily measurable process variables (referred to as “auxiliary variables”) and key target variables that are difficult to measure (referred to as “primary variables”) [4,5]. Such soft-sensor models effectively emulate the functionality of hardware sensors used to predict primary variables, unaffected by the complex environments of industrial processes, and inherently possess advantages in terms of universality, flexibility, and cost-effectiveness [6,7].

Soft-sensor methods are commonly categorized into data-driven and knowledge-driven methods [8]. Due to challenges in accurately establishing knowledge-driven models for complex industrial manufacturing processes, data-driven soft-sensor methods are currently mainstream [9,10]. Compared to traditional machine learning-based soft-sensor methods, deep learning-based soft-sensor methods have proven to achieve higher accuracy in most cases [11,12,13]. However, these high-accuracy soft-sensor models are not widely applied because their security and reliability face serious threats from adversarial attacks [14,15].

Deep neural networks are highly susceptible to imperceptible adversarial attacks, resulting in error outputs with high confidence [16,17]. DLSS models also face serious threats from adversarial attacks. Firstly, from an external environment perspective, an increasing number of complex industrial sites have control systems that are highly interconnected to corporate and public networks, making it easy for attackers to exploit various software and hardware vulnerabilities to infiltrate industrial control systems and carry out adversarial attacks on deployed DLSS models [18]. Secondly, from the structural characteristics of the models, DLSS itself is based on deep neural networks, which are naturally vulnerable to adversarial attacks. In recent years, some studies have proposed specific adversarial attack methods targeting DLSS. For instance, a time-series generator is proposed to implement adversarial attacks on DLSS [19]. In addition, an adversarial attack method, namely iterative directly attack output (IDAO), is proposed, and experimental attacks on the industrial ammonia synthesis process have been conducted [20]. The experimental results indicate that fake prediction results obtained by adversarial attacks could lead to misjudgments about the current process state and cause erroneous actions by actuators or mistakes by engineers, such as unnecessarily reducing material input and incorrectly lowering reaction temperatures, endangering normal industrial production and potentially causing severe safety accidents.

Therefore, it is crucial to research adversarial defense methods for DLSS to enhance the model’s ability to defend against adversarial attacks and establish robust soft-sensor models that are resilient to such attacks. Adversarial training is the primary method for enhancing the adversarial robustness of deep learning models, recognized as one of the most effective adversarial defense methods [21]. The samples generated from normal samples subjected to adversarial attacks are called adversarial samples. Adversarial training aims to enhance the adversarial robustness of DLSS models by utilizing these adversarial samples for training, a process formally considered as performing min-max optimization. The internal maximization problem involves finding adversarial samples that cause maximum loss to the deep learning model—solving this problem corresponds to the process of adversarial attacks, where the obtained optimal adversarial samples are considered to possess the most powerful attack capability. The external minimization problem involves finding deep neural network parameters that minimize the loss due to such adversarial attacks [22]. Clearly, to improve the adversarial robustness of the DLSS model through adversarial training, both the external minimization problem and the internal maximization problem of adversarial training need to be better solved.

To solve the internal maximization problem, it is essential to propose more powerful adversarial attack methods to obtain stronger adversarial samples. Currently, most attack methods targeting DLSS employ black-box transfer attacks. Since attackers typically lack knowledge of the structure and parameters of the original soft sensor (OSS), they generate adversarial samples by attacking a constructed proxy soft sensor (PSS) model. These adversarial samples with transferability are then used to attack the OSS model to achieve the black-box transfer attacks. The challenge with this attack strategy lies in estimating the transfer gradient on the PSS (the gradient used to execute black-box transfer attacks, referred to as the transfer gradient). The accuracy of this transfer gradient estimation ultimately determines the efficacy of the attack. Results from black-box transfer attacks demonstrated that the obtained adversarial samples achieved excellent attack effects on the PSS [18,20]. However, when transferred to attack the OSS, the rationality of the fake prediction results decreased and their fluctuation significantly increased, making the attack more easily detectable and hampering its effectiveness. Most current black-box transfer attack methods greedily utilize gradient directions to update adversarial samples, which impacts the accurate estimation of the transfer gradient. Consequently, adversarial samples often fall into bad local minima, resulting in the inability to generate stronger adversarial samples. The momentum method, an optimization technique that memorizes historical gradient information, can be introduced into the process of adversarial attacks on DLSS to achieve a more accurate estimation of the transfer gradient and warrants further research.

For solving external minimization problems, it is necessary to address the challenge of adversarial robustness overfitting. This issue arises when the model becomes highly susceptible to overfitting adversarial samples after adversarial training, leading to a decrease in sensing accuracy on the original normal samples [23]. Studies on adversarial training for DLSS have shown that the model inevitably suffers from adversarial robustness overfitting. After adversarial training, DLSS often struggles to balance both adversarial robustness and sensing accuracy [24]. Authors have explored similar studies on adversarial training methods for DLSS, combining the knowledge-guided adversarial attack (KGAA) method with normal adversarial training for defense and obtaining similar experimental results [18]. Therefore, further research is needed to develop new adversarial training methods that mitigate the loss of DLSS accuracy by effectively addressing the overfitting problem of adversarial robustness. Domain adaptation has been proven effective in eliminating domain distribution differences as representative approaches in transfer learning, thereby better capturing common features between source and target domains [25,26]. Therefore, it treats the target domain as that of adversarial samples and the source domain as that of the original sample, then carries out soft-sensor adversarial training under the framework of domain adaptive training to learn the common information of the adversarial samples and the original samples, thus preventing the occurrence of adversarial robust overfitting.

In summary, the security of DLSS faces significant challenges from adversarial attacks, necessitating the enhancement of the model’s adversarial robustness through adversarial training. However, the problems of transfer gradient estimation and robust overfitting in soft-sensor adversarial training have not been adequately addressed. Therefore, the soft sensor for quality variables during the growth of semiconductor silicon single crystals serves as a case study to investigate adversarial training defense methods for DLSS. The main contributions of this article are as follows:

(1) To address the challenge of accurately estimating transfer gradients, historical gradient information is integrated into the iterative process of generating an adversarial sample. This integration leverages the similarity of gradients between adjacent iteration steps, stabilizing the update direction and enhancing the accuracy of transfer gradient estimation. Consequently, this assists the iterative process in converging to adversarial samples with stronger transferability.
(2) To mitigate the overfitting issue in adversarial training, a soft-sensor DAAT model was developed within the domain adversarial training framework. This model aims to learn common features shared between adversarial and original samples, thereby achieving a balance between sensing accuracy and adversarial robustness, while avoiding excessive learning bias toward either side.
(3) The effectiveness of the proposed DLSS adversarial training method is validated in a typical complex interconnected industrial process of silicon single-crystal growth manufacturing. This study aims to draw attention to and promote further investigation into adversarial defense methods for DLSS, facilitating the safe and reliable deployment of DLSS in complex industrial processes.

The remainder of this article is structured as follows: Section 2 introduces the proposed adversarial training method, Section 3 presents a comparison and analysis of experimental results, and Section 4 concludes the study.

## 2. Materials and Methods

### 2.1. Proposed Adversarial Training for DLSS

**Figure 1:**
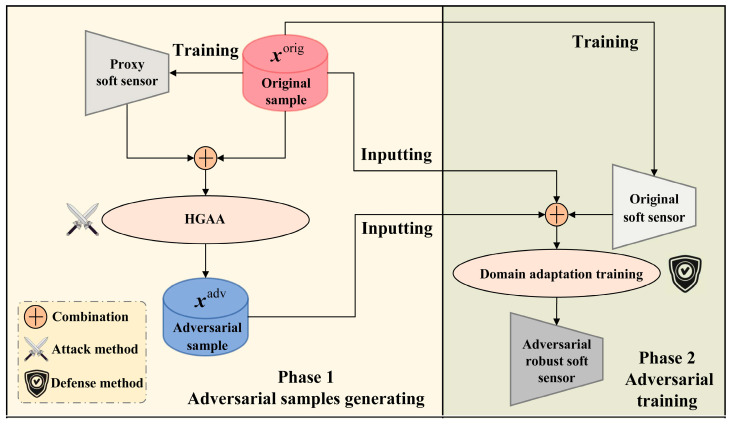
Domain-adaptation adversarial training method.

To enhance the adversarial robustness of DLSS models, a new adversarial training, named domain-adaptation adversarial training (DAAT), is proposed. As shown in Figure 1, DAAT primarily consists of two stages: the first stage involves generating adversarial samples, and the second involves domain-adaptation training based on these adversarial samples. It is important to note that this article focuses on adversarial defense, with both stages discussed from the defender’s perspective. In the first stage, tasks typically performed by attackers are considered. This involves data hijacking on the transmission link to build a PSS based on the acquired data, followed by launching a black-box transfer attack. From the defender’s perspective, this operation translates into training a PSS with accuracy close to that of the OSS using existing samples and then applying the attack algorithm to generate adversarial samples. The first stage of DAAT can utilize different adversarial attack methods. In this study, a historical gradient-based adversarial attack (HGAA) is proposed for generating adversarial attacks. Therefore, the default attack method discussed in this paper is HGAA. The HGAA proposed in the first stage and the DLSS domain-adaptive training method proposed in the second stage will be detailed in Section 2.2 and Section 2.3.

### 2.2. HGAA for DLSS

Since the KGAA effectively addresses scenarios where sample labels cannot be obtained during attacks, the attack model of HGAA is constructed based on the KGAA method as follows:

(1) $$\max_{\delta }G((x^{\mathrm{orig}}+\delta ),r;\theta ),$$

$$s.t.x^{\mathrm{orig}}\;\in \;S,$$

(2) $${\left\|\delta \right\|}_{\infty }\;\leq \;\varepsilon ,$$

where $G((x^{\mathrm{orig}}+\delta ),r;\theta )=-L(y_{\mathrm{KDSS}},f(x^{\mathrm{orig}}+\delta ;\theta ))-\mathrm{rB}(x^{\mathrm{orig}}+\delta )$; herein, $G((x^{\mathrm{orig}}+\delta ),r;\theta )$ represents the loss function, f represents the data-driven PSS model, θ represents the parameter of the PSS model, δ represents the imperceptible perturbations generated by adversarial attacks (the $l_{\infty }$-norm of δ models the imperceptibility), $x^{\mathrm{orig}}$ represents the original input data (i.e., samples not yet attacked), $x^{\mathrm{orig}}+\delta$ represents the adversarial samples, $f(x^{\mathrm{orig}}+\delta ;\theta )$ represents the output of the PSS model after the attack, $B(x^{\mathrm{orig}}+\delta )\;=1/f(x^{\mathrm{orig}}+\delta )-y_{\mathrm{KDSS}}$, $y_{\mathrm{KDSS}}$ represents the predicted value of the knowledge-driven soft sensor model corresponding to the process, L represents the mean-square error loss function, r is a very small positive number, and S represents the feasible domain.

Unlike the KGAA method, which greedily uses gradient directions to update adversarial samples [18], HGAA introduces the concept of the momentum method into the adversarial attack process for DLSS. It adaptively corrects the update direction by establishing the correlation between the gradient at the current moment and the transfer gradient at the historical moment. Specifically, in the *n*-th iteration of HGAA, the transfer gradient of the loss function with respect to the input samples is calculated as follows:

(3) $$g_{n+1}=\nabla_{x_{n}}G((x^{\mathrm{orig}}+\delta ),r;\theta ).$$

The correlation *α* between the adjacent transfer gradients $g_{n}$ and $g_{n+1}$ is determined by the cosine similarity *β* and the distance similarity ξ, calculated as:

(4) $$\beta =\cos(g_{n},g_{n+1})=\left\{\begin{array}{c} 1,n=0\\ \frac{g_{n}\cdot g_{n+1}}{{\left\|g_{n}\right\|}_{2}\cdot {\left\|g_{n+1}\right\|}_{2}},n\neq 0 \end{array}\right.,$$

(5) $$\xi ={\left\|g_{n}-g_{n+1}\right\|}_{2},$$

(6) α=0.5 × β+0.5 × ξ.

A stronger correlation between the gradients of two adjacent iterations results in a larger α, indicating minor changes in the gradient direction. Conversely, less correlation between gradients of two consecutive iterations results in a smaller α, suggesting more reliance on the gradient of the previous iteration to reduce errors in the update direction. The weighted gradient is calculated using the following equation, and the resulting gradient is used as the final estimate of the transfer gradient in the *n*-th iteration:

(7) $$g_{n+1}=\alpha g_{n+1}+(1-\alpha )g_{n},$$

The momentum item is then updated based on the transfer gradient obtained at this time:

(8) $$m_{n+1}=\mu m_{n}+\frac{g_{n+1}}{{\left\|g_{n+1}\right\|}_{1}},$$

where μ represents the momentum decay coefficient. Due to changes in the gradient values during iterations, the weighted gradient $g_{n+1}$ is normalized by dividing its norm, and the adversarial sample at the *n*-th iteration is calculated based on the obtained momentum item:

(9) $$x_{n+1}^{\mathrm{adv}}=x_{n}+\eta \mathrm{sign}(m_{n+1}),$$

where sign represents the signum function and η represents the perturbation size added for each iteration. When the number of iterations *n* reaches the preset number $N_{1}$, the iteration stops, and the adversarial samples $x^{\mathrm{adv}}$ are generated from the original input samples $x^{\mathrm{orig}}$.

### 2.3. Domain Adaptation Training for DLSS

**Figure 2:**
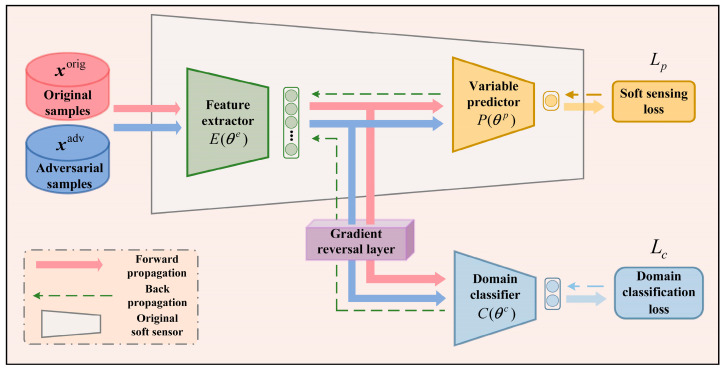
Structure of the domain-adaptive training model for soft sensing.

The DAAT method utilizes adversarial samples $x^{\mathrm{adv}}$ generated by HGAA for training an adversarial robust DLSS model through domain adaptation. The structure of the proposed domain-adaptive training model is shown in Figure 2, where *E* represents a feature extractor, *P* is a primary variable predictor, and *C* is a domain classifier, all implemented using deep neural networks with parameters denoted as $\theta^{e}$, $\theta^{p}$, and $\theta^{c}$, respectively. The original DLSS model can be divided into *E* and *P* structurally, where *E* and *P* together constitute the DLSS model. The initial parameters of both are inherited from the trained OSS. During domain-adaptation training, the parameter $\theta^{p}$ remains unchanged, while $\theta^{e}$ and $\theta^{c}$ are the parameters to be learned. The optimization objectives are:

(10) $$\underset{\theta^{c}}{\mathrm{argmin}}L_{C},$$

(11) $$\underset{\theta^{e}}{\mathrm{argmin}}L_{P}-\lambda L_{C},$$

where λ represents the scaling factor for the reversed gradients, $L_{P}$ represents the sensing loss, and $L_{C}$ represents the domain classifier loss. The computational equations for these losses are defined as:

(12) $$L_{P}=\sum_{x_{i}^{\mathrm{orig}},y_{i}^{\mathrm{orig}}\in D^{\mathrm{orig}}}\mathrm{MSE}(P(E(x_{i}^{\mathrm{orig}})),y_{i}^{\mathrm{orig}})+\;\;\;\;\;\;\;\;\;\;\;\;\;\;\;\;\;\;\;\;\;\;\;\;\;\;\;\;\;\;\;\;\;\;\;\;\;\;\;\;\;\;\;\;\;\;\;\;\;\;\;\;\;\;\;\;\;\;\;\;\;\;\;\;\;\;\;\;\;\;\;\;\;\;\;\;\sum_{x_{i}^{\mathrm{adv}}\in D^{\mathrm{adv}},y_{i}^{\mathrm{orig}}\in D^{\mathrm{orig}}}\mathrm{MSE}(P(E(x_{i}^{\mathrm{adv}})),y_{i}^{\mathrm{orig}}),$$

(13) $$L_{C}=\sum_{x_{i}^{\mathrm{orig}}\in D^{\mathrm{orig}}}\mathrm{BCE}(C(E(x_{i}^{\mathrm{orig}})),0)+\sum_{x_{i}^{\mathrm{adv}}\in D^{\mathrm{adv}}}\mathrm{BCE}(C(E(x_{i}^{\mathrm{adv}})),1),$$

where MSE represents the mean-square error and BCE represents the binary cross entropy [22]. During training, both original samples $x_{i}^{\mathrm{orig}}$ and adversarial samples $x_{i}^{\mathrm{adv}}$ are processed by the feature extractor *E*, followed by primary variable prediction using *P* and domain classification using the gradient reversal layer and domain classifier *C*. The gradient reversal layer does not affect the forward propagation of data but reverses gradients during backpropagation to help *E* extract common features between original and adversarial samples. Under the constraints of $L_{P}$ and $L_{C}$, the common features extracted by *E* make it challenging for C to distinguish between original and adversarial samples, while minimizing the sensing loss, thus allowing the OSS to balance both adversarial robustness and predictive performance through DAAT. The domain-adaptive training model uses the gradient descent algorithm for iterative training. In the *n*-th iteration, the updates for the parameters $\theta^{e}$ and $\theta^{c}$ are computed as follows:

(14) $$\theta_{n+1}^{e}=\theta_{n}^{e}-\zeta (\frac{\partial L_{P}}{\partial \theta_{n}^{e}}-\lambda \frac{\partial L_{C}}{\partial \theta_{n}^{e}}),$$

(15) $$\theta_{n+1}^{c}=\theta_{n}^{c}-\zeta \frac{\partial L_{C}}{\partial \theta_{n}^{c}},$$

where ζ represents the learning rate. The domain-adaptive training stops when the predefined number of iterations $N_{2}$ is reached. Finally, *E* and *P* are extracted and sequentially connected to obtain a DLSS model with adversarial robustness after DAAT.

## 3. Experiments

### 3.1. Case Description

**Figure 3:**
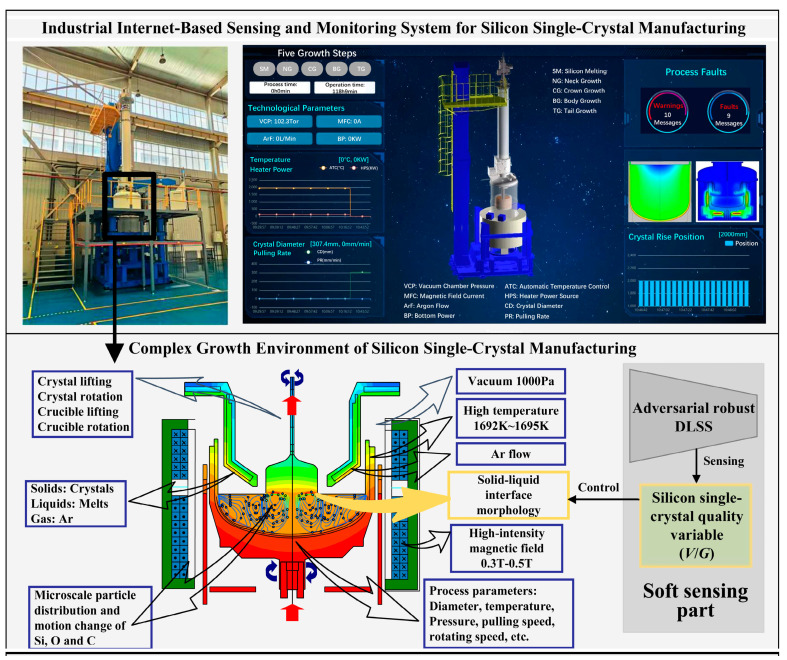
Complex industrial manufacturing process of silicon single-crystal growth.

This paper explores an adversarial defense method for DLSS using a semiconductor soft sensor to measure quality variables during silicon single-crystal manufacturing as a case study, as illustrated in Figure 3. The manufacturing process of silicon single crystals takes place in an environment characterized by high temperatures, inert gases, and strong magnetic fields, making it a complex industrial manufacturing process. To minimize the formation of native point defects during silicon single-crystal growth and achieve isodiametric growth, the crystal quality variable (denoted as *V*/*G*, where *V* represents the lifting speed of the crystal and *G* represents the temperature gradient of the crystal growth interface) is sensed and controlled to maintain it within a normal range. This ensures that the solid–liquid interface morphology remains ideal, resulting in the growth of high-quality silicon single crystals [27].

Due to the challenges faced by hardware sensors in stably measuring *V*/*G* in complex and harsh environments over extended periods, DLSS was developed to predict *V*/*G*. Eight auxiliary variables including crystal diameter, main heater power, crystal rising speed, crucible rising speed, heating element temperature, liquid surface temperature, crystal rotation speed, and crucible rotation speed were obtained, with *V*/*G* serving as the primary variable. As part of the ongoing establishment of a silicon single-crystal growth digital twin monitoring system based on an industrial internet platform, the production environment transitioned from a closed system to an interconnected one with the outside world. This placed stringent requirements on the deployed DLSS to defend against adversarial attacks effectively. Strong adversarial robustness in the DLSS model ensures that the soft sensor results of *V*/*G* remain unaffected by adversarial attacks, thus controlling *V*/*G* within the normal range and ensuring the quality of crystal manufacturing. Therefore, soft-sensor data from the silicon single-crystal growth process were used to establish the DLSS model, and the proposed DAAT method was employed to enhance the DLSS’s adversarial robustness. Specific parameter settings for the experiment will be detailed in the following subsection.

### 3.2. Parameter Settings

The DAAT method consists of two stages: the generation of adversarial samples and domain-adaptive training. Therefore, the parameter settings for the adversarial sample generation stage were established first. The OSS model structure was defined as 8-64-48-32-16-10-1. A PSS model, which achieved prediction accuracy close to that of the OSS, was trained with a structure of 8-64-32-16-1. The OSS and PSS are all constructed with fully connected layers. The activation function for the hidden neurons in the OSS and PSS is set to ReLU. Before training the OSS and PSS, the connection weights of the two models are initialized via the Xavier method [28]. The training dataset size for OSS and PSS was set to 10,000, the validation dataset to 2000, and the testing dataset to 400 (specifically allocated for evaluating adversarial training results). For convenience, model inputs are normalized to [0, 1]. Both models were trained using the Adam optimizer with default parameter settings and training halted upon reaching the early stopping condition [29]. The mechanism-driven soft sensor model used in the KGAA method is detailed in [30]. Next, the HGAA parameters were empirically set as follows: $N_{1}=20$, r=0.05, η=0.0025, and μ=0.55. The parameter settings for the domain-adaptation training stage primarily involved the structures of the feature extractor *E*, primary variable predictor *P*, and domain classifier *C*, set as 8-64-48-32, 32-16-10-1, and 32-24-18-9-1, respectively. Other parameters were empirically set as follows: λ=1, ζ=0.001, and $N_{2}=1000$. 

### 3.3. Results

**Figure 4:**
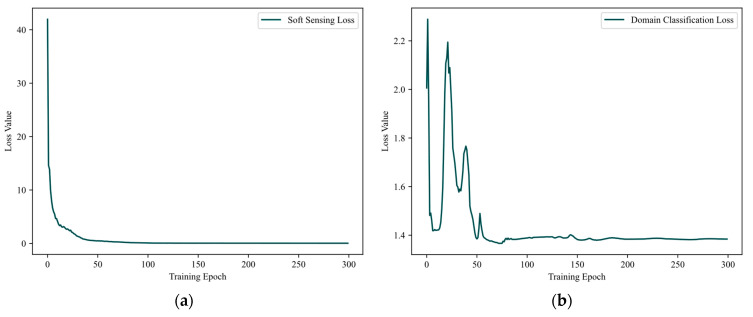
Convergence process of DAAT. (**a**) Soft sensing loss. (**b**) Domain classification loss.

First, the domain-adaptive training model must be confirmed to be well-trained. The change curve of the model’s loss function is illustrated in Figure 4. In an ideal case, after DAAT, E in the model can extract the common features of the adversarial samples and the original samples, while C cannot effectively distinguish the two. Because BCE was selected when calculating the domain classification loss L_C_, the ideal loss value of L_C_ should approximate convergence to 1.38 after quantizing C’s output to [0, 1] [31]. DAAT training stops when it reaches the early stopping condition, when the value of L_C_ eventually converges around 1.38, as depicted in Figure 4a. Meanwhile, the blackish-green curve in Figure 4b indicates that the soft sensing loss L_P_ decreases continuously during the DAAT process, indicating that the prediction accuracy of the soft sensor model is constantly improving. Combining the changes in the two curves confirms that the domain-adaptive training model has been well-trained after DAAT. The features extracted by E not only make it difficult for C to distinguish between normal and adversarial samples but also consider the soft sensing prediction accuracy.

**Figure 5:**
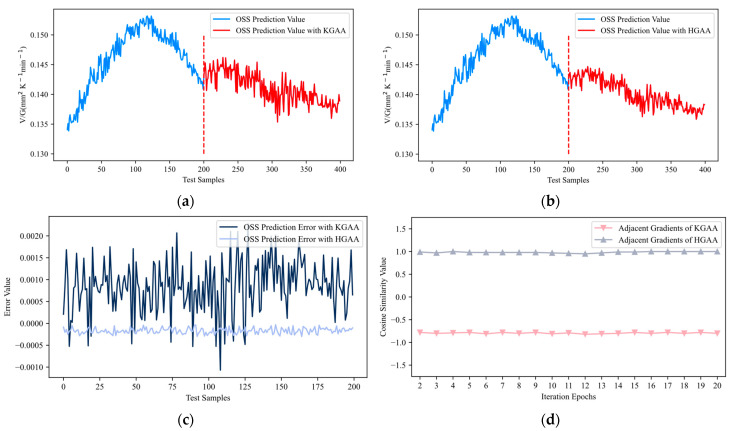
Attack results. (**a**) Attack effect of KGAA. (**b**) Attack effect of HGAA. (**c**) OSS prediction error. (**d**) Cosine similarity of adjacent gradients.

Next, a comparison of the attack efficacy between KGAA and the proposed method HGAA was conducted on the test dataset, with the results of transfer attacks from both methods depicted in Figure 5. The first half curve in blue represents the output of 200 unattacked original samples. After the second half’s 200 adversarial samples are fed into OSS, the prediction output is represented by the red curve (these 200 adversarial samples were obtained by KGAA and HGAA, respectively). The output fluctuations after being attacked by KGAA are notably distinct, showing a significant deviation from normal predictions. In contrast, the output fluctuations after being attacked by HGAA depict less change, aligning more closely with the output of the unattacked samples. Such an attack is considered more feasible in practical applications.

In the soft-sensing phase of industrial manufacturing, operators are often unaware of the true label value of the current input sample. However, they understand domain knowledge and possess the mechanism-driven soft sensor model. Therefore, by comparing the output value of the current sample with that of the mechanism-driven model, it is possible to determine whether the current output is reasonable. The error curves between the output after adversarial attacks (KGAA and HGAA) and the output of the mechanism-driven model are plotted in Figure 5c. The error values between the output values after HGAA and the output of the mechanism-driven model are small. In contrast, the error values between the output values after KGAA and the output of the mechanism-driven model are large. Meanwhile, the mean absolute error (MAE) values between the outputs after the attacks (HGAA and KGAA) and the mechanism model’s output were calculated as 0.2 × 10^−3^ and 0.9 × 10^−3^, respectively. The output attacked by HGAA exhibited a smaller MAE, indicating that HGAA was a more rational attack method numerically. Therefore, HGAA is considered closer to the operator’s understanding.

The key difference between HGAA and KGAA lies in introducing historical gradients to correct the update direction in HGAA, enabling a more accurate estimation of the transfer gradient. As illustrated in Figure 5d, in the iterative process of HGAA and KGAA, the cosine similarity curves between adjacent gradients are grey and pink, respectively. Each point on the curves is averaged by the cosine similarity of 200 test samples. A cosine similarity of 1 indicates that the adjacent gradients are pointing in the same direction, and a cosine similarity of −1 indicates that the adjacent gradients are pointing in opposite directions. The cosine similarity of adjacent gradients of HGAA is approximately 1 at most points, which indicates that the direction of adjacent gradients of HGAA can remain similar in the iterative process. This enables the algorithm to stabilize in one direction for more accurate searches during the adversarial sample generation phase. The cosine similarity of the adjacent gradients of KGAA is between −0.5 and −0.1 in most cases, indicating certain differences in the direction of the two adjacent gradients in the iterative process. Compared with HGAA, KGAA lacks the operation of introducing historical gradients to correct the update direction, which makes KGAA unable to rely on the gradient of the previous iteration to reduce the error in the update direction during the iteration process, thus making it difficult for the algorithm to carry out accurate searches in a stable direction. Based on the analysis results shown in Figure 5 and numerical metrics, HGAA demonstrated superior performance. Therefore, it was concluded that HGAA obtained stronger adversarial samples by achieving a more accurate estimation of transfer gradients, making it a more powerful attack method.

**Figure 6:**
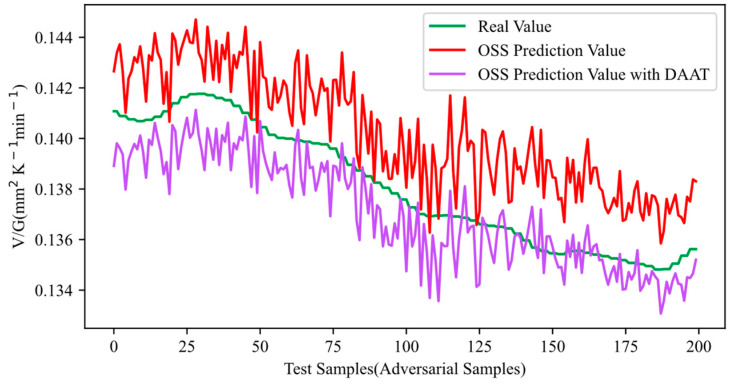
Adversarial robustness testing results after DAAT (inputting adversarial samples).

**Figure 7:**
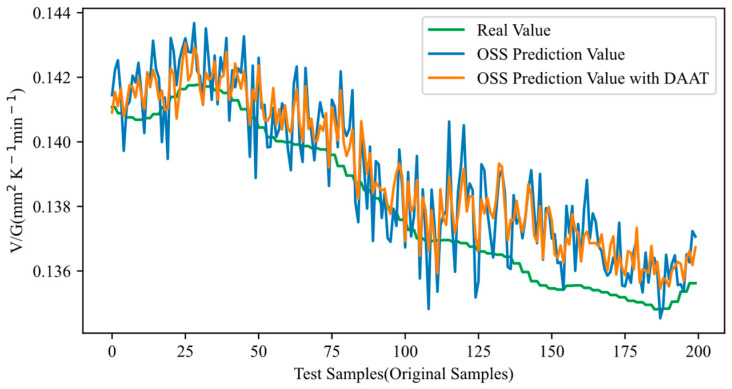
Adversarial robustness testing results after DAAT (inputting original samples).

Adversarial samples were generated for the set of 10,000 original training samples using HGAA, and DAAT was conducted using these generated adversarial samples. After completing DAAT, the last 200 out of 400 test samples were input to evaluate the adversarial robustness of the OSS, and the results are shown in Figure 6 and Figure 7. In Figure 6, the adversarial samples are fed into the OSS trained with DAAT, and the resulting output is represented by the purple curve. It is noticeable that the output curve shifts closer to the real curve after DAAT, indicating a reduction in the error between the output and the actual values. The bias introduced by the adversarial samples was mitigated, demonstrating that DAAT effectively defended against HGAA and enhanced the adversarial robustness of the OSS. Figure 7 illustrates the performance of the OSS trained with DAAT when original samples are input. The resulting output is represented by the tan-colored curve. The fluctuations in the output curve reduced, and the curve shifted closer to the real curve after DAAT. This reduction in fluctuations signifies a decrease in prediction errors, indicating an improvement in prediction accuracy for the original samples. In conclusion, the DAAT method not only enhanced adversarial robustness but also led to a slight improvement in prediction accuracy, achieving the desired effect.

**Figure 8:**
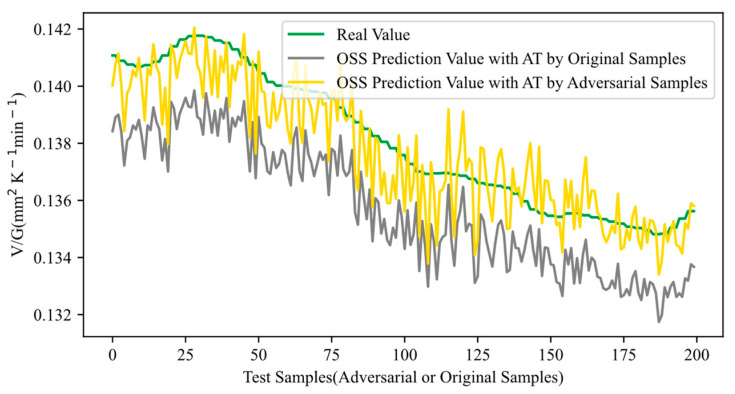
Adversarial robustness testing results after NAT (inputting adversarial or original samples).

The adversarial robustness test results of the normal adversarial training (NAT) method are illustrated in Figure 8. The NAT also includes two phases: the first stage is adversarial sample generation, and the second stage is adversarial training. The adversarial samples utilized in NAT are generated by HGAA, and the sample number and parameter settings are exactly the same as HGAA in DAAT. In other words, the first stage of NAT and the first stage of DAAT are the same, but their second stages differ. The NAT adopts classical adversarial training methods, and the training process and settings are consistent with those in [32], while DAAT employs the proposed domain-adaptive training method. In Figure 8, the gray curve represents the OSS prediction results after the NAT with original samples, while the yellow curve depicts predictions with adversarial samples. As observed in Figure 8, the yellow curve closely approximates the real curve, whereas the gray curve exhibits a noticeable deviation from the real curve. This indicates that the OSS model, after NAT, has overlearned from adversarial samples, resulting in decreased accuracy when predicting with original samples and demonstrating significant adversarial robust overfitting. Comparing this with the DAAT method, represented by the purple and yellow curves in Figure 6 and Figure 7, respectively, both curves are distributed around the truth curve and closely approximate it. This suggests that the OSS model, following DAAT, did not overlearn from either adversarial or original samples. In conclusion, compared to NAT, DAAT effectively mitigates the issue of adversarial robust overfitting and achieves a better balance between original prediction accuracy and adversarial robustness.

To further demonstrate the effectiveness of the proposed method, contrast experiments were conducted, and the numerical results are presented in Table 1. In the table, $x^{\mathrm{orig}}$ represents the original samples, $x^{\mathrm{adv}\_\mathrm{HGAA}}$, $x^{\mathrm{adv}\_\mathrm{IDAO}}$, $x^{\mathrm{adv}\_\mathrm{RNAA}}$, and $x^{\mathrm{adv}\_\mathrm{KGAA}}$ represent the adversarial samples generated by $x^{\mathrm{orig}}$ using the HGAA, IDAO, RNAA, and KGAA methods, respectively, MAE represents the prediction error of OSS, ${\mathrm{MAE}}^{\mathrm{DAAT}\_\mathrm{HGAA}}$ and ${\mathrm{MAE}}^{\mathrm{DAAT}\_\mathrm{KGAA}}$ represent the prediction error of OSS after applying DAAT (with the HGAA and KGAA attack methods, respectively), and ${\mathrm{MAE}}^{\mathrm{NAT}\_\mathrm{HGAA}}$ represents the prediction error of OSS after NAT using adversarial samples generated by HGAA.

Table 1 illustrates that when the input is $x^{\mathrm{orig}}$, MAE is ${1.2\times 10}^{-3}$, with ${\mathrm{MAE}}^{\mathrm{DAAT}\_\mathrm{HGAA}}$ decreasing to ${1.0\times 10}^{-3}$. Conversely, ${\mathrm{MAE}}^{\mathrm{DAAT}\_\mathrm{KGAA}}$ and ${\mathrm{MAE}}^{\mathrm{NAT}\_\mathrm{HGAA}}$ increase, suggesting that DAAT based on HGAA can maintain good prediction accuracy when dealing with original samples. The difference between DAAT based on HGAA and DAAT based on KGAA lies in HGAA’s more accurate estimation of the transfer gradient, which aids in generating superior adversarial samples and improving OSS predictive performance. When the input is $x^{\mathrm{adv}\_\mathrm{HGAA}}$, the MAE is ${3.1\times 10}^{-3}$, and ${\mathrm{MAE}}^{\mathrm{DAAT}\_\mathrm{HGAA}}$, ${\mathrm{MAE}}^{\mathrm{DAAT}\_\mathrm{KGAA}}$, and ${\mathrm{MAE}}^{\mathrm{NAT}\_\mathrm{HGAA}}$ all exhibit varying degrees of decline. This indicates that these three adversarial training methods enhance defense against HGAA. Among the three MAE values, the value of ${\mathrm{MAE}}^{\mathrm{DAAT}\_\mathrm{HGAA}}$ is the lowest. When the input is $x^{\mathrm{adv}\_\mathrm{KGAA}}$ and $x^{\mathrm{adv}\_\mathrm{RNAA}}$, the ${\mathrm{MAE}}^{\mathrm{DAAT}\_\mathrm{HGAA}}$ is also the lowest, and when the input is $x^{\mathrm{adv}\_\mathrm{IDAO}}$, the ${\mathrm{MAE}}^{\mathrm{DAAT}\_\mathrm{HGAA}}$ is the second lowest, indicating that the DAAT method based on HGAA defends against HGAA and also achieves good defense against KGAA, IDAO, and RNAA. When the input is $x^{\mathrm{adv}\_\mathrm{IDAO}}$, the value of ${\mathrm{MAE}}^{\mathrm{DAAT}\_\mathrm{HGAA}}$ is bigger than the value of ${\mathrm{MAE}}^{\mathrm{DAAT}\_\mathrm{KGAA}}$. It does not mean that the DAAT method based on KGAA improves the overall robustness of OSS. The experimental results only confirm that the OSS has a stronger defense capability against IDAO at this time. As IDAO in this example exhibits a significant shift in attack results, the practicality of the attack is insufficient, and it is difficult to better simulate real attack behavior. Therefore, it is more practical to employ the HGAA, KGAA, and RNAA methods to evaluate the defense ability of OSS because the three attack methods are commonly considered practical attack methods. In summary, the DAAT method based on HGAA balances against adversarial samples while maintaining predictive accuracy for original samples, thereby enhancing the adversarial robustness of DLSS models.

### 3.4. Discussion

Although adversarial training can enhance a model’s adversarial robustness, the effectiveness of using NAT to improve the adversarial robustness of the DLSS model (i.e., the OSS model in the experiment) is quite limited. The issues of transfer gradient estimation and overfitting in adversarial robustness often arise, yet previous relevant research has not systematically addressed these problems. Therefore, this paper proposes a novel adversarial training method, DAAT, to tackle these challenges in DLSS, yielding promising results. While many effective adversarial training methods have been developed for classification models, DLSS as a regression prediction model is still at an early stage of improving its adversarial robustness. The proposed method represents a valuable exploration in this area. In addition, most studies in the DLSS field select representative industrial process data and verify the effectiveness of the proposed method on the process data. The data size is generally between thousands and tens of thousands, and the data dimension (the number of auxiliary variables) is between several to dozens. Therefore, this study is consistent with the convention of most studies, utilizing data from the V/G soft-sensing process with a similar data size as most studies to verify the effectiveness of the proposed DAAT method. In future studies, datasets with larger sizes will be utilized to further enhance the method’s performance. Meanwhile, the method will also be validated on data from other cases to improve potential limitations in the results.

## 4. Conclusions

To address the challenges associated with the accurate estimation of transfer gradients and adversarial robustness overfitting when employing adversarial training methods to enhance the adversarial robustness of DLSS, a new DLSS adversarial training method named DAAT was proposed and its effectiveness was verified through the soft-sensing stage of silicon single-crystal quality variables. During the DAAT process, HGAA was employed, which introduced historical gradient information into the iterative process of generating adversarial examples. This approach considers the similarity of gradients between adjacent iteration steps to stabilize the update direction, thereby increasing the accuracy of transfer gradient estimation and generating stronger adversarial examples. This optimization addresses the internal maximization problem in DAAT. DLSS learns the common features between adversarial samples generated by HGAA and original samples through DAAT, achieving a good balance between sensing accuracy and adversarial robustness. This optimization addresses the external minimization problem in DAAT. As a result, DLSS not only enhances its adversarial robustness against HGAA but also acquires defensive ability against KGAA after undergoing DAAT. Given that DLSS is deployed in safety-critical industrial environments, merely defending against existing attack methods is insufficient. It is essential to further study more robust adversarial training methods to defend against potential new types of unknown adversarial attacks. Additionally, developing detection methods for adversarial attacks is crucial to quickly distinguish between normal samples and adversarial samples, thus establishing a more efficient and comprehensive defense system. Ultimately, improving the safety and reliability of DLSS and promoting its extensive deployment and application in complex industrial processes requires advancements in both adversarial training methods and adversarial attack detection techniques. This holistic approach will contribute to the overall security and reliability of DLSS in critical industrial settings.

## Figures and Tables

**Table 1:** Adversarial robustness testing results for different adversarial training methods.

Test Samples	Test Indexes			
	MAE	MAE^DAAT_HGAA^	MAE^DAAT_KGAA^	MAE^NAT_HGAA^
$x^{\mathrm{orig}}$	${1.2\times 10}^{-3}$	${1.0\times 10}^{-3}$	${1.4\times 10}^{-3}$	${3.4\times 10}^{-3}$
$x^{\mathrm{adv}\_\mathrm{HGAA}}$	${3.1\times 10}^{-3}$	${1.1\times 10}^{-3}$	${1.5\times 10}^{-3}$	${1.7\times 10}^{-3}$
$x^{\mathrm{adv}\_\mathrm{KGAA}}$	${4.1\times 10}^{-3}$	${1.1\times 10}^{-3}$	${1.5\times 10}^{-3}$	${1.4\times 10}^{-3}$
$x^{\mathrm{adv}\_\mathrm{IDAO}}$	${11.4\;\times \;10}^{-3}$	${4.4\;\times \;10}^{-3}$	${3.1\;\times \;10}^{-3}$	$\;{5.6\;\times \;10}^{-3}$
$x^{\mathrm{adv}\_\mathrm{RNAA}}$	${3.2\;\times \;10}^{-3}$	${3.1\;\times \;10}^{-3}$	${3.6\;\times \;10}^{-3}$	${\;4.0\;\times \;10}^{-3}$

## Data Availability

Data are available for reasonable reasons by contacting the corresponding author.
